# The diversified defocus profile of the near‐work environment and myopia development

**DOI:** 10.1111/opo.12698

**Published:** 2020-06-09

**Authors:** Kai Yip Choi, Angela Yuen‐ting Mok, Chi‐wai Do, Paul Hong Lee, Henry Ho‐lung Chan

**Affiliations:** ^1^ The Centre for Myopia Research School of Optometry The Hong Kong Polytechnic University Kowloon Hong Kong; ^2^ School of Nursing The Hong Kong Polytechnic University Kowloon Hong Kong

**Keywords:** home size, living environment, myopia, near work

## Abstract

**Purpose:**

To quantify the defocus characteristics in the near‐work environment at home and investigate the relationship with subsequent myopia progression.

**Methods:**

Fifty subjects (aged 7–12 years) were recruited and followed for 1 year. The home near‐work environment (writing desk) was measured at a baseline home‐visit using the Kinect‐for‐Windows to capture a 3‐dimensional image. The depth values of the image were then converted into scene defocus with respect to the subject’s viewpoint. The defocus characteristics were quantified as the dioptric volume (the total amount of net defocus, or DV) and standard deviation of the defocus values (SD_D_). Information on home size, time spent outdoors, and in front of a desk were also obtained. Univariate correlation, and multivariate regression were used to assess the association between myopia progression, defocus characteristics, and other co‐variates.

**Results:**

The baseline spherical equivalent refraction (*M*) and refraction change over 1 year (∆*M*) were − 1.51 ± 2.02 D and − 0.56 ± 0.45 D respectively. DV was not significantly correlated with ∆*M* (Spearman’s ρ = −0.25, *p* = 0.08), while SD_D_ was negatively correlated to ∆*M* (Spearman’s ρ = −0.42, *p* = 0.003). Although SD_D_ was not a significant predictor in multivariate analysis, the regional DV at 15°–20° eccentricity was significant (*p* = 0.001). Home size (*F*_2,50_ = 7.01, *p* = 0.002) and time spent outdoors (Independent *t* = −2.13, *p* = 0.04) were also associated with ∆*M*, but not time spent in front of desk (Independent *t* = 0.78, *p* = 0.44).

**Conclusion:**

The defocus profile in the home environment within the para‐central field of view is associated with childhood refractive error development.

## Introduction

Over the past decades, the prevalence of myopia has escalated in developed countries.^1^ This rapid increase has been linked to environmental effects, which are critical for refractive error development.^2^ In East and South‐east Asian countries, the high prevalence of myopia has been attributed to the intense levels of near work during school age. Studies have quantified near work in terms of working distance,^3^, ^4^ time span,^5^, ^6^ type of near work,^3^, ^7^ and weighted near work (i.e. dioptre hour).^8^, ^9^ However, the relationship between near work and myopia remains controversial.^10^

Visual input at the peripheral retina has also been suggested to regulate eye growth. Animal studies have shown that despite absence of foveal integrity, peripheral visual signals could mediate central refractive development.^11^, ^12^ Peripheral hyperopic and myopic defocus were found to cause myopic and hyperopic changes, respectively.^12^ This phenomenon is reflected in the success of myopia control in terms of manipulating defocus over most of the retina using orthokeratology,^13^ multifocal contact lenses,^14^ and defocus‐incorporated spectacle lenses.^15^ However, it remains controversial whether the myopia control effect emanates from the peripheral myopic defocus induced by these devices,^16^ as several studies showed that initial peripheral refractive errors in children were not associated with the incidence or onset of myopia.^17^, ^18^ In addition, other designs of myopia control intervention targeting reduction of peripheral hyperopia, failed to effectively control refractive development in children.^19^ Although it is generally accepted that the peripheral retina plays a role in ocular growth, the relationship between peripheral refractive error and myopia development still needs further investigation.

In daily life, human eyes are exposed simultaneously to myopic and hyperopic defocus from the environment.^20^ Based on the simultaneous defocus concept, Flitcroft simulated human visual scenes using customised computer software.^21^ In this simulation, outdoor scenes have a more evenly‐distributed dioptric profile, while indoor scenes (within an office) have an uneven dioptric profile. The distribution of the defocus is even more varied when indoor object distance is closer. The uneven distribution of peripheral defocus from the indoor environment was suggested as a myopia risk factor for children, especially those who spent less time outdoors, to have a higher incidence and prevalence of myopia.^21^, ^22^ In a previous study, it was reported that small home size in Hong Kong was associated with more myopia and longer axial length.^23^ The reason for this increased risk was suggested to be the peripheral hyperopic defocus due to the close surroundings in small homes. However, it is still unknown whether certain home environment characteristics, for example, reading desk scenes, other than home size, could contribute to children’s refractive development.

In Hong Kong and elsewhere in East‐Asia, children spend many hours in near work at home to complete their heavy load of school work.^24^, ^25^ It is important to understand how the home‐working environment, especially for near tasks, affects their refractive errors. Myopia studies investigating near tasks have focused mainly on the type of visual task, while the details of the visual scene, for example, the dioptric profile, received little attention. With the emergence of depth sensing technology,^26^, ^27^ it is possible to obtain more information (e.g. depths across the visual field) from a scene with a handy device.^28^, ^29^ In the current study, it was aimed to quantify the amount of relative scene defocus in a near‐work environment, and investigate the relationship between such environments and juvenile refractive development.

## Methods

### Subjects and ocular data

Fifty‐nine healthy Chinese subjects aged between 7 and 12 years were recruited in the Optometry Clinic of The Hong Kong Polytechnic University to participate in the study from December 2016 to October 2017. Six subjects were excluded because they started orthokeratology (*n* = 1), had a major home renovation (*n* = 1), or were lost to follow‐up (*n* = 4) within the study period. An additional three subjects were excluded because they had a glass‐surfaced working desk, creating specular reflection, preventing measurement of the dioptric profile. Informed consent and simple written assent were obtained from the parent and the subject, respectively. The study protocols were approved by the Human Subjects Ethics Subcommittee of The Hong Kong Polytechnic University and all procedures followed the tenets of the Declaration of Helsinki. Subjects attended an initial eye examination in the clinic and a second follow‐up a year later. Cycloplegic refraction, using an open‐field autorefractor (NVision K5001, http://www.shin‐nippon.jp/products/nvk5001/), was measured 30 min after instilling two drops of 1% cyclopentolate five minutes apart. Five measurements were taken and the value was transposed into spherical equivalent refraction (*M*) using the following equation:

$$M=S+\frac{C}{2},$$

where *S* is the spherical error and *C* is the cylindrical error.

The change in *M* (∆*M*) in the right eye after 1 year was analysed.

### Home visit and visual scene measurement

Each subject’s home was visited to capture the daily visual scene before the baseline eye examination. As children in Hong Kong spend hours on school work,^24^, ^25^ their reading desk (for near tasks) was chosen as the target visual scene. The daily time in front of the desk, as well as weekly time outdoors, were reported by the subject and confirmed by the parent/guardian. Subjects were asked to perform some near work sitting in their usual position at the desk, which was confirmed by their parent/guardian. The desk was presented in its usual format, with the subject’s own homework exercise book in place. The distance from the visual target (a book) to the eye position was measured using a metric tape. The photograph of home scene was shown to the parent/guardian at the 1‐year follow up visit to confirm there was no major change of furniture position.

**Figure 1:**
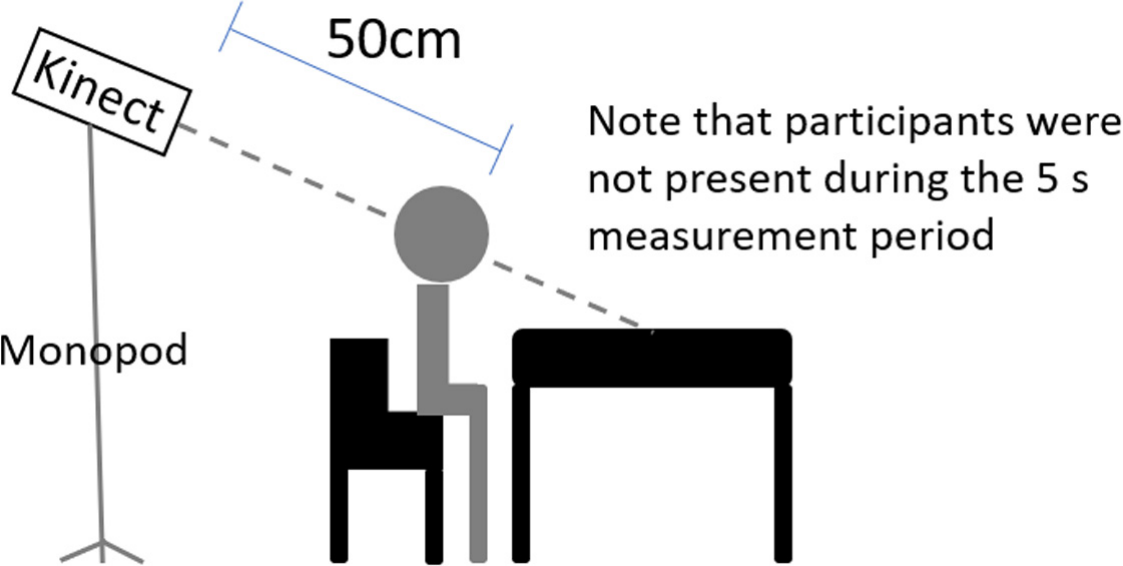
Schematic diagram of the measurement setup.

To obtain the information of the visual scene, Kinect‐for‐Windows v2 (Hardware discontinued in 2017, software development kit available on https://www.microsoft.com/en‐hk/download/details.aspx?id=44561) was used to capture the scene depth in the first visit.^26^, ^27^ It consists of an infra‐red emitter and an infra‐red camera, which allow the device to capture the scene depth by calculating the time of flight of the infra‐red light ray. Before the measurement, subjects were first asked to take up their usual working posture, which was confirmed by their parent/guardian. Because of the working range of the device (0.5 m–4.5 m), the Kinect was set 50 cm behind the subject’s eye position. The Kinect was directed to point at the visual target (e.g. a book) and aligned with the subject’s line of sight on a monopod to maintain stability. Subjects were then asked to leave the scene and the scene depth was captured for at least 5 s at a frequency of 1 Hz, i.e. a total of at least five depth images were obtained (*Figure* *1*). The Kinect depth images, which were superimposed to obtain the average values, consisted of 512 × 434 pixels carrying a distance value corresponding to the points in the scene from the device.

### Data processing and analysis

As the Kinect was placed behind the subject, the values were corrected by calibrating the central pixel value according to the actual viewing distance measured by the metric tape. The depth values were inversed to dioptres, and then calibrated with respect to the centre of the visual target, e.g. a book on the desktop, which was set as zero. Hence, the points, which were further away than the visual target, would be of negative dioptric value (myopic defocus), while those closer than the visual target would be of positive dioptric value (hyperopic defocus). Finally, the visual scene was reconstructed using the dioptric depth values across vertical and horizontal dimensions, i.e. a 3‐dimenional dioptric space,^30^ with respect to the subject’s viewpoint. If there was any window within the measured field, this part of the scene would be regarded as no vergence (i.e. distant with respect to the central visual target). One of the limitations of the current study was the lack of eye fixation data, in which the scene defocus could not be mapped with the retinal defocus. Another limitation was the lack of continuity of data acquisition, in which baseline data was obtained.

**Figure 2:**
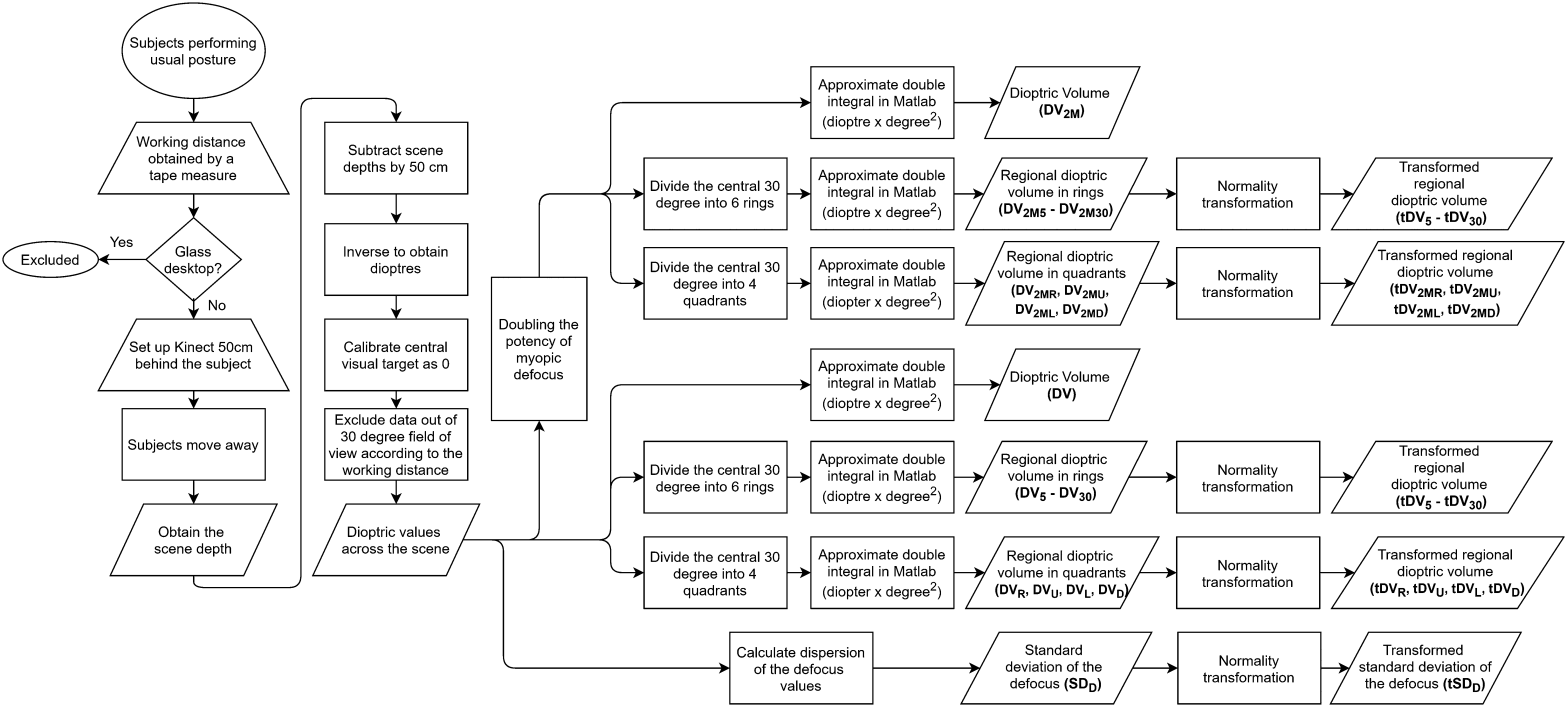
Flow chart for defocus data acquisition and processing.

To represent the overall dioptric power of the visual scene, the dioptric volume (DV) of the scene was defined as the approximate double integrals computed by *trapz* function in MATLAB (www.mathworks.com) over the central 30° field of view (i.e. dioptre × degree^2^, or D°°). The DV was calculated based on both linear (assuming the positive and negative powers cancelled each other out) and non‐linear (assuming myopic defocus was twice potent of hyperopic defocus, i.e. DV_2M_)^20^ relationships. Central 30° field of view was chosen because this could accommodate all subjects with different working distances within the device’s working angle of view. In simple terms, the dioptric volume represented the total amount of net defocus generated from the scene with respect to the central visual target. In addition, to assess the overall dispersion of the scene dioptric defocus, the standard deviation of the scene defocus (SD_D_) was also calculated. A flow‐chart summarizing the procedures is shown in *Figure* *2*. The partial correlation between DV and ∆*M*, and between SD_D_ and ∆*M* were calculated using Spearman’s test, with baseline M as the confounding factor.

**Figure 3:**
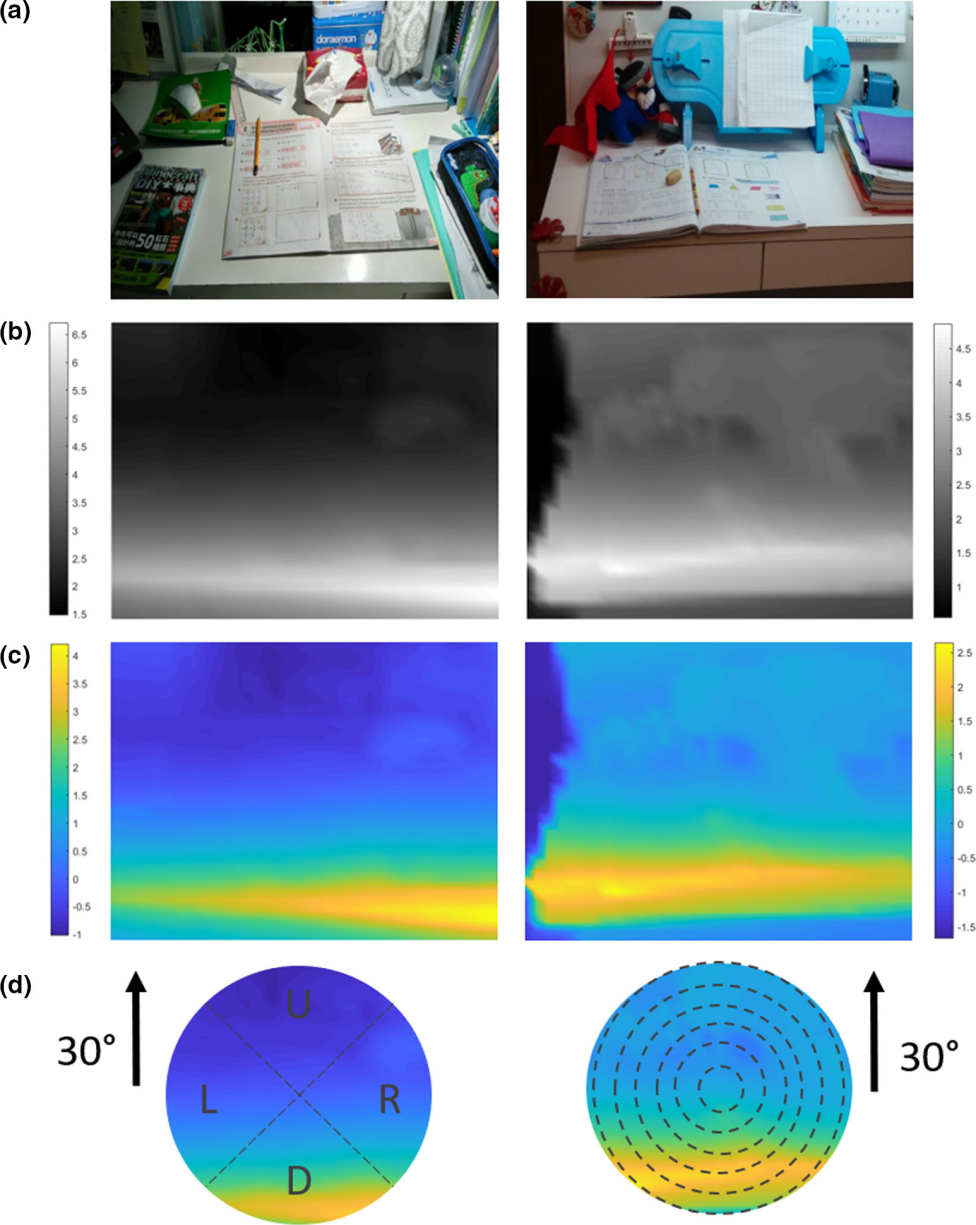
Scene demonstration of subjects’ desks. (a) Coloured picture. (b) Dioptric (inversed distance) map. (c) Scene defocus map after calibration. (d) Analysis region of central 30°. This region was divided into six rings and four quadrants respectively for secondary analysis. Positive defocus indicates hyperopic while negative defocus indicates myopic defocus. Colour scale in dioptres.

To assess the regional effect, the central 30° field of view was divided into six rings of 5° interval and four quadrants (right, up, left, and down) respectively (*Figure* *3*). The DV was calculated in each ring and quadrant to evaluate the effect of eccentricity and location of the scene respectively. To simplify the statistics, the regional DVs were transformed to achieve normality using a two‐step transformation^31^ (Before transformation: K‐S tests > 0.18, *p* < 0.001; after transformation: K‐S tests < 0.04, *p *> 0.20). The correlation between transformed dioptric volume in each ring (i.e. tDV_5_ to tDV_30_) and ∆*M*, and between quadrant (i.e. tDV_R_, tDV_U_, tDV_L_, and tDV_D_) and ∆*M* were individually calculated using Spearman’s test, and then entered into multiple linear regression models, as well as other factors, including age, baseline *M*, time spent in front of desk, time spent outdoors, working distance, parental myopia (by self‐reporting), home size, and transformed SD_D_ (tSD_D_), to predict the 1‐year ∆*M* of the subject. The stepwise removal method was further used to cater for our small sample size in the regression models, which was to eliminate insignificant variables starting from the one with the highest *p* value, until the *p* values of all remaining variables were below 0.05.

It was previously reported that home size was associated with the childhood refractive error of Hong Kong children. In the current study, the relationship between home size and ∆*M*, as well as the DV and SD_D_ were investigated. Subjects were sequenced according to their home sizes and were categorised into three groups: Small (*N* = 16, Range 297–500 ft^2^/27.6 – 46.5 m^2^), Medium (*N* = 17, Range 503–602 ft^2^/46.7–5.9 m^2^), and Large (*N* = 17, Range 614–1400 ft^2^/57.0–130.1 m^2^). One‐way analysis of variance (or Kruskal‐Wallis test) was used to compare the ∆*M*, DV, and SD_D_, with Bonferroni post‐hoc test, among the three home size groups. The ∆*M* was also compared among parental myopia using one‐way analysis of variance with Bonferroni post‐hoc test. In addition, the daily time spent in front of the desk and weekly time spent outdoor (transportation excluded) were classified as Low [<2 h/day^24^ and <2 h/week (median), respectively] and High (≥2 h/day and ≥2 h/week) respectively, with which the ∆*M* were then compared using separate independent *t*‐tests. Only the data from the right eye was analysed. Significance level was set at 0.05 and all tests were performed with SPSS Statistics V22.0 software (https://www.ibm.com/analytics/spss‐statistics‐software).

## Results

The subjects (*n* = 50) in the study were aged 9.3 ± 1.2 years (Mean ± S.D.) and had a baseline refraction of −1.51 ± 2.02 D. The ∆*M* was −0.56 ± 0.45 D over 1 year. The median daily time subjects spent in front of the desk was 2 h/day (IQR 1.0–3.0 h/day, Range 0.5–5.0 h/day), with an average working distance of 29.7 ± 6.0 cm, while the median weekly time spent outdoor was 2 h/week (IQR 1.0–4.5 h/week, Range 1.0–20.0 h/week). With respect to the scene defocus parameters, the median DV and DV_2M_ over the central 30° were 1.16 D°° (IQR 0.46–3.82 D°°, Range −0.48–8.43 D°°) and 0.86 D°° (IQR 0.27 to 3.56 D°°, Range −1.36 to 8.24 D°°), while the median SD_D_ over the central 30° was 0.49 D°° (IQR 0.31–0.69 D°°, Range 0.08–2.29 D°°).

The working distance of the subjects was not related to the ∆*M* (Pearson’s *R* = 0.21, *p* = 0.15), but was negatively correlated with DV (Spearman’s ρ = −0.60, *p* < 0.001) and SD_D_ (Spearman’s ρ = −0.67, *p* < 0.001) respectively. Thus, the shorter the working distance, the more positive and dispersed the overall scene defocus. For the partial correlation controlling for the baseline M, DV (Spearman’s ρ = −0.25, *p* = 0.08) and DV_2M_ (Spearman’s ρ = −0.21, *p* = 0.16) were not related to ∆*M*, while SD_D_ was negatively correlated to ∆*M* (Spearman’s ρ = −0.42, *p* = 0.003), i.e. subjects with faster myopia progression had a more dispersed baseline scene defocus.

Among the regional defocus, DV_20_ was significant correlated with ∆*M* (Spearman’s ρ = −0.32, *p* = 0.02) but no quadrant DV nor DV_2M_ was correlated. *Table* *1* shows the statistical results of other correlations. Multiple regression analyses revealed that only age, baseline *M*, and tDV_20_ were significantly associated with ∆*M* in the regression models. Tables S1–S4 list the detailed statistical analyses of individual variable. The results from the stepwise regression models showed that older children and those having more hyperopic baseline M had significantly slower myopia progression. On the other hand, more hyperopic para‐central defocus at 15°–20° (i.e. tDV_20_ and tDV_2M20_) and left quadrant (i.e. tDV_L_ and tDV_2ML_) from the scene were associated with faster myopia progression. The coefficients and statistics of the significant variables are listed in *Table* *2*.

**Table 1:** Correlation between regional defocus and refractive change over 1 year

	DV_5_	DV_10_	DV_15_	DV_20_	DV_25_	DV_30_
Spearman’s ρ	−0.12	−0.12	−0.23	−0.32†	−0.13	0.04
*p* Value	0.42	0.40	0.11	0.02	0.37	0.78

	DV_R_	DV_U_	DV_L_	DV_D_
Spearman’s ρ	−0.18	0.27	−0.22	−0.18
*p* value	0.21	0.06	0.12	0.22

	DV_2M5_	DV_2M10_	DV_2M15_	DV_2M20_	DV_2M25_	DV_2M30_
Spearman’s ρ	−0.15	−0.08	−0.23	−0.22	−0.07	0.11
*p* value	0.30	0.56	0.12	0.12	0.63	0.47

	DV_2MR_	DV_2MU_	DV_2ML_	DV_2MD_
Spearman’s ρ	−0.16	0.26	−0.19	−0.15
*p* value	0.27	0.06	0.20	0.31

DV, dioptric volume; D, Down; L, Left; R, Right; U, Up. 2M indicates 2× myopic defocus potency.

† Indicates a significance level of < 0.05.

**Table 2:** Stepwise multiple regression on refractive change over 1 year

	Raw B value	95% CI	Standardised *B* value	*p* Value	VIF
1× myopic defocus potency ring analysis: Adjusted *R* ^2^ = 0.32, *F*_3,50_ = 8.63, *p* < 0.001					
Age	0.12	0.03 to 0.29	0.31	0.01	1.02
Baseline M	0.05	0.01 to 0.11	0.24	0.05	1.01
tDV_20_	−0.18	−0.28 to −0.08	−0.43	0.001	1.03
1× myopic defocus potency quadrant analysis: Adjusted *R* ^2^ = 0.18, *F*_3,50_ = 4.58, *p* = 0.01					
One myopic parent	−0.38	−0.68 to −0.07	−0.34	0.02	1.10
tDV_U_	0.14	0.02 to 0.26	0.31	0.03	1.10
tDV_L_	−0.17	−0.28 to −0.05	−0.40	0.01	1.11
2× myopic defocus potency ring analysis: Adjusted *R* ^2^ = 0.31, *F*_3,50_ = 8.18, *p* < 0.001					
Age	0.12	0.03 to 0.21	0.32	0.01	1.02
Baseline M	0.06	0.01 to 0.12	0.28	0.03	1.04
tDV_2M20_	−0.18	−0.28 to −0.07	−0.42	0.001	1.05
2× myopic defocus potency quadrant analysis: Adjusted *R* ^2^ = 0.16, *F*_4,50_ = 4.09, *p* = 0.01					
Medium home size	0.26	0.01 to 0.51	0.28	0.04	1.04
High time spent outdoors	0.27	0.04 to 0.50	0.31	0.02	1.04
tDV_2ML_	−0.28	−0.58 to −0.00	−0.26	0.05	1.05
tSD_D_	−0.13	−0.25 to 0.02	−0.31	0.02	1.08

tDV, transformed dioptric volume; tSD_D_, transformed standard deviation of the defocus; R, Right; U, Up; L, Left; D, Down. 2M indicates 2× myopic defocus potency.

The univariate analyses are listed in *Table* *3*. Home size was associated with ∆*M* (One‐way ANOVA, *F*_2,50_ = 7.01, *p* = 0.002), but not with DV (Kruskal‐Wallis test, χ^2^_2,50_ = 0.40, *p* = 0.82), DV_2M_ (Kruskal‐Wallis test, χ^2^_2,50_ = 0.44, *p* = 0.80), or SD_D_ (Kruskal‐Wallis test, χ^2^_2,50_ = 3.81, *p* = 0.15). In post‐hoc tests, children living in a Small‐sized home had greater myopia progression than those in a Medium‐sized (Bonferroni post‐hoc test, *p* = 0.02) and Large‐sized home (Bonferroni post‐hoc test, *p* = 0.003). No significant association was found between Medium‐ and Large‐sized homes (Bonferroni post‐hoc test, *p *> 0.99). Parental myopia was not significantly associated with ∆*M* (One‐way ANOVA, *F*_2,50_ = 2.44, *p* = 0.10). There was no significant difference between the Low and High groups (Independent *t* = 0.78, *p* = 0.44) for daily time spent in front of their desk. With respect to the weekly time spent outdoors, the Low group progressed significantly faster than the High group (Independent *t* = −2.13, *p* = 0.04), but, neither the correlation between time spent outdoors and scene defocus (DV: Spearman’s ρ = −0.24, *p* = 0.10; SD_D_: Spearman’s ρ = −0.15, *p* = 0.30) nor the correlation between daily time spent in front of desk and scene defocus (DV: Spearman’s ρ = 0.13, *p* = 0.36; SD_D_: Spearman’s ρ = 0.15, *p* = 0.29) reached significance.

**Table 3:** Univariate analyses on myopia progression over 1 year

	*N*	∆*M* (Mean ± S.D.)
Total	50	−0.56 ± 0.45 D
Home size		
Small home	16	−0.87 ± 0.52 D†
Medium home	17	−0.46 ± 0.32 D†
Large home	17	−0.38 ± 0.35 D†
Parental myopia		
No myopic parent	6	−0.23 ± 0.43 D
One myopic parent	21	−0.67 ± 0.52 D
Two myopic parents	23	−0.55 ± 0.35 D
Time spent in front of desk		
Low (<2.0 h daily)	21	−0.50 ± 0.47 D
High (≥2.0 h daily)	29	−0.61 ± 0.43 D
Time spent outdoors		
Low (<2.0 h weekly)	24	−0.70 ± 0.47 D§
High (≥2.0 h weekly)	26	−0.44 ± 0.40 D§

† ‡ Significant difference in Bonferroni *post‐hoc* test.

^§^ Significant difference in independent *t*‐test.

## Discussion

The current study revealed an association between children’s home‐working environment and their subsequent refractive development. Specifically, the defocus profile from the scene, in terms of the dispersion of defocus distribution and para‐central regional defocus, was associated with the myopic change in refractive error in a year. As reported previously, small home size was not only associated with more myopic refractive error, but also a risk factor for faster myopic change.

**Figure 4:**
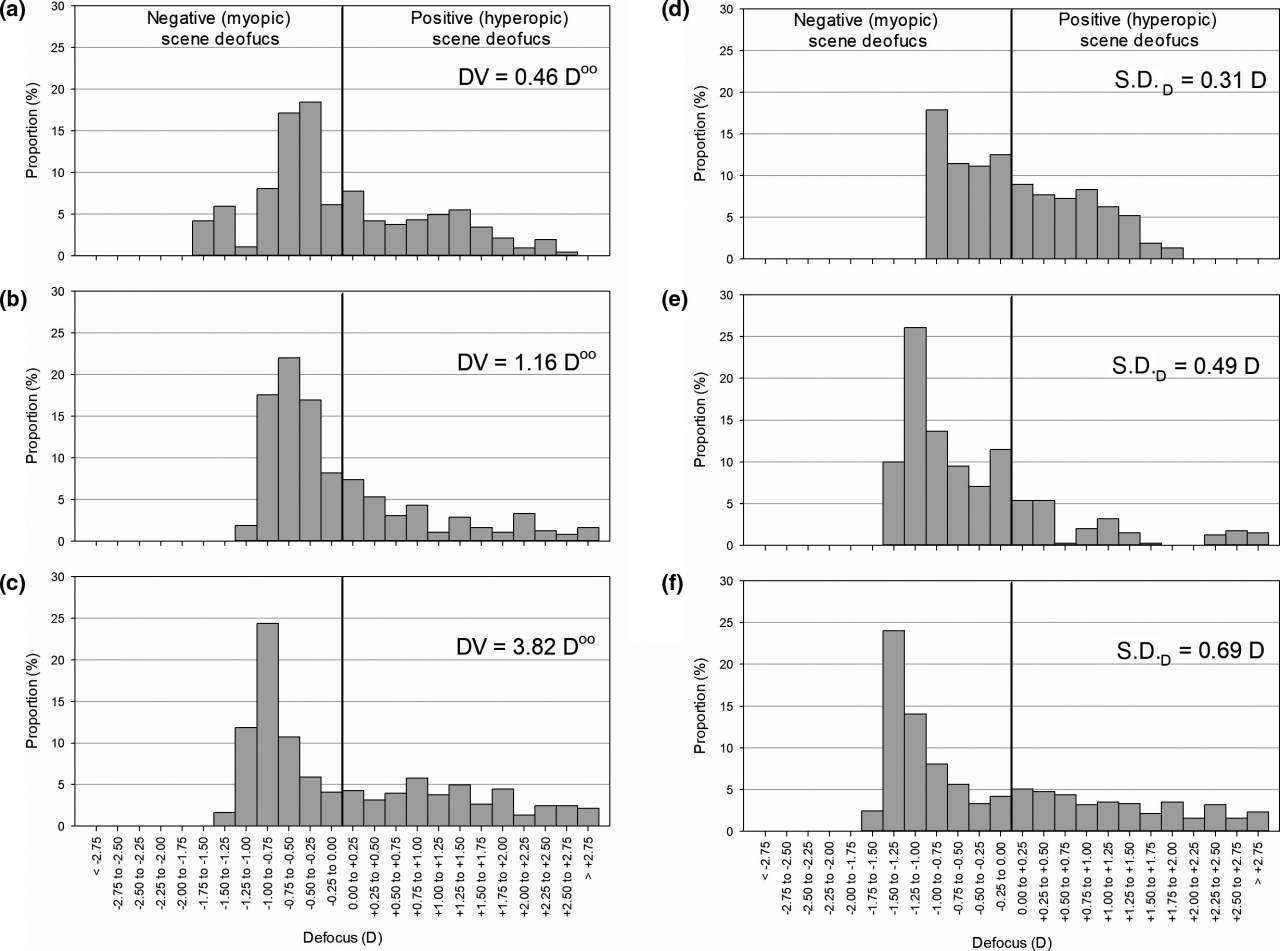
Scene defocus distribution of central 30° from representative subjects – Subject a: The 1st quartile of dioptric volume (DV); Subject b: The 2nd quartile of DV; Subject c: The 3rd quartile of DV; Subject d: The 1st quartile of standard deviation of the scene defocus (SD_D_); Subject e: The 2nd quartile of SD_D_; Subject f: The 3rd quartile of SD_D_. The DV represented the total amount of net defocus, while the SD_D_ represented the dispersion of scene defocus value within the central 30°.

Findings for adverse effects of near work on childhood refractive development are controversial.^10^ In the current study, a novel quantification of a near work environment was devised. Garcia and co‐workers described their measurements to capture the defocus map using an older version of Kinect (v1) and an eye tracker^29^ by overlapping the acquired frames from both devices over five minutes. Unlike the computer working desk in their study, the ‘in‐focus’ area of a child’s writing/reading desk did not show a maximum (*Figure* *4*) because the desk surface was inclined with respect to the eyes. Instead, most of the area of view incorporated a range of negative defocus. However, the calculated DVs, which is the total amount of net defocus of the central 30° field, for most subjects were positive, because the magnitude of the positive defocus was generally greater than that of negative defocus. *Figure* *4* shows the representative distributions (quartiles) regarding DV and SD_D_, which is the dispersion of scene defocus value within the central 30°.

These findings suggest a moderate correlation between peripheral defocus dispersion and myopia progression. Unlike an outdoor scene, in which peripheral defocus distribution is more uniform, an indoor scene consists of a more varied defocus profile, creating a rapid change in peripheral retinal defocus when the fixation is changed. Such rapid change has been suggested to cause a failure in emmetropization as the temporal integration of the retinal signals is interrupted,^21^, ^22^ in which this non‐linear temporal integration was demonstrated in studies performed in both guinea pigs and monkeys.^32^, ^33^ With respect to spatial integration, myopic defocus demonstrated approximately twice the potency of hyperopic defocus when simultaneously presented in chicks.^20^ In the current study, this doubled myopic potency assumption could not better explain the relationship between scene defocus and myopic progression, which may be due to the difference in species as the myopic potency appeared less prominent in mammals.^34^ In addition, as in an earlier study conducted by our group, a smaller home was associated with more myopic refractive error.^23^ We speculated a smaller home would create more surrounding hyperopic defocus, as the constricted environment would block the view of distant objects and/or have more close objects.

In contrast, the total amount of net defocus within the central 30° field (i.e. DV and DV_2M_) was not associated with refractive error development. Instead, the amount of net defocus across 15°–20° eccentricity had a modest, but significant correlation with the change in refractive error as well as the multiple linear regression analysis controlled for other covariates. Unfortunately, it was not possible to match the scene defocus with the subjects’ fixation to generate the retinal defocus map as illustrated by Garcia *et al*.^35^ However, the para‐central retina has been reported to have higher defocus sensitivity in electroretinography studies.^36^, ^37^ If the scene eccentricity is matched with the retinal eccentricity, the positions of the closely surrounding objects near the central visual target is likely to manipulate the peripheral retinal signal in control of the emmetropization process. However, quadrant analysis did not show any conclusive association with myopia progression. Among the four quadrants, the left quadrant was significantly associated with DV in multivariate analyses, but not univariate analyses. A possible explanation could be the writing habit due to the dominance of right laterality, but further study is necessary to determine if this is the case.

To our knowledge, the current study is the first to investigate home environmental defocus in relation to children’s refractive error development. Yet, there are several limitations preventing a comprehensive interpretation of the results. Firstly, the defocus profile measurement was performed at a single time point instead of longitudinally incorporating observation of changes in the environment, meaning that only the subsequent refractive change throughout the year with the baseline environmental defocus could be observed. Secondly, the DV was calculated as a net defocus over the 3‐dimenional space, assuming the positive and negative defocus would equally cancel each other out. Finally, as the subjects were required to move away during the measurement, such a move would ignore the defocus created by the subjects’ own body parts, e.g. arms placed on the desk. In future studies, handy smartphones with duo, or even trio‐camera could be used to gather longitudinal data at a larger scale. In addition, eye trackers could have been incorporated into the measurement to map the environmental defocus onto the retinal defocus,^35^ as well as to investigate the defocus temporal integration,^32^, ^33^, ^38^ to evaluate myopia development.

## Conclusion

The defocus profile in the home environment, especially within the para‐central field of view, is associated with childhood refractive error development and is likely a potential myopia risk factor.

## Conflict of interest

The authors report no conflicts of interest and have no proprietary interest in any of the materials mentioned in this article.

## Supporting information


**Table S1.** Multiple regression with all variables on refractive change over 1 year by ring analysis.
**Table S2.** Multiple regression with all variables on refractive change over 1 year by quadrant analysis.
**Table S3.** Multiple regression with all variables with doubled myopic defocus potency on refractive change over 1 year by ring analysis.
**Table S4.** Multiple regression with all variables with doubled myopic defocus potency on refractive change over 1 year by quadrant analysis.
**Table S5.** Descriptive statistics of the regional dioptric volume.
