# Supplementary material for: The diversified defocus profile of the near‐work environment and myopia development
Source: Ophthalmic Physiol Opt. 2020 Jun 9;40(4):463–71. doi: 10.1111/opo.12698 (PMC7497190; doi:10.1111/opo.12698)
Supplement: Supplementary file 1 — Table S1. Multiple regression with all variables on refractive change over 1 year by ring analysis. Table S2. Multiple regression with all variables on refractive change over 1 year by quadrant analysis. Table S3. Multiple regression with all variables with doubled myopic defocus potency on refractive change over 1 year by ring analysis. Table S4. Multiple regression with all variables with doubled myopic defocus potency on refractive change over 1 year by quadrant analysis. Table S5. Descriptive statistics of the regional dioptric volume. [file OPO-40-463-s001.docx]

Supplementary table 1. Multiple regression with all variables on refractive change over 1 year by ring analysis.

| Adjusted R^2^ = 0.38, F_16,50_ = 2.88, p = 0.01 | | | | | |
| --- | --- | --- | --- | --- | --- |
|  | Raw B value | 95% CI | Standardised  B value | p value | VIF |
| **Age** | **0.23*** | **0.01 to 0.44** | **0.54** | **0.04** | **1.62** |
| **Baseline M** | **0.09*** | **0.02 to 0.16** | **0.40** | **0.01** | **1.86** |
| Working distance | 0.01 | -0.02 to 0.04 | 0.13 | 0.52 | 3.01 |
| Time in front of desk | -0.11 | -0.24 to 0.01 | -0.28 | 0.08 | 1.95 |
| Parental myopia (ref no myopic parent) | | | | | |
| One myopic parent | -0.19 | -0.52 to 0.13 | -0.18 | 0.23 | 1.62 |
| Two myopic parents | -0.23 | -0.51 to 0.05 | -0.26 | 0.10 | 1.85 |
| Home size (ref Small) |  |  |  |  |  |
| Medium | 0.15 | -0.14 to 0.44 | 0.16 | 0.30 | 1.82 |
| Large | 0.23 | -0.07 to 0.52 | 0.24 | 0.13 | 1.93 |
| Time spent outdoors | 0.23 | -0.03 to 0.49 | 0.26 | 0.08 | 1.59 |
| tDV_5_ | 0.03 | -0.08 to 0.14 | 0.09 | 0.53 | 5.13 |
| tDV_10_ | -0.10 | -0.35 to 0.14 | -0.25 | 0.39 | 6.66 |
| tDV_15_ | 0.03 | -0.24 to 0.29 | 0.06 | 0.84 | 7.75 |
| **tDV_20_** | **-0.19*** | **-0.37 to -0.00** | **-0.46** | **0.05** | **3.98** |
| tDV_25_ | 0.03 | -0.21 to 0.28 | 0.08 | 0.78 | 6.80 |
| tDV_30_ | 0.08 | -0.09 to 0.26 | 0.21 | 0.34 | 3.71 |
| tSD_D_ | -0.10 | -0.32 to 0.13 | -0.23 | 0.39 | 5.32 |
|  |  |  |  |  |  |
| tDV: transformed dioptric volume  tSD_D_: transformed standard deviation of scene defocus  * indicated significance of p < 0.05 | | | | | |

Supplementary table 2. Multiple regression with all variables on refractive change over 1 year by quadrant analysis.

| Adjusted R^2^ = 0.20, F_14,50_ = 1.86, p = 0.07 | | | | | |
| --- | --- | --- | --- | --- | --- |
|  | Raw B value | 95% CI | Standardised  B value | p value | VIF |
| Age | 0.03 | -0.10 to 0.16 | 0.09 | 0.63 | 1.80 |
| Baseline M | 0.06 | -0.02 to 0.13 | 0.25 | 0.13 | 1.52 |
| Working distance | 0.00 | -0.03 to 0.03 | 0.03 | 0.91 | 2.64 |
| Time in front of desk | -0.13 | -0.40 to 0.14 | -0.14 | 0.34 | 1.34 |
| Parental myopia (ref no myopic parent) | | | | | |
| One myopic parent | -0.33 | -0.68 to 0.03 | -0.30 | 0.07 | 1.50 |
| Two myopic parents | 0.11 | -0.18 to 0.39 | 0.12 | 0.44 | 1.50 |
| Home size (ref Small) |  |  |  |  |  |
| Medium | 0.26 | -0.05 to 0.57 | 0.28 | 0.10 | 1.58 |
| Large | -0.01 | -0.30 to 0.29 | -0.01 | 0.96 | 1.47 |
| Time spent outdoors | 0.24 | -0.04 to 0.51 | 0.27 | 0.09 | 1.39 |
| tDV_R_ | 0.07 | -0.21 to 0.36 | 0.17 | 0.61 | 6.84 |
| tDV_U_ | 0.10 | -0.05 to 0.24 | 0.22 | 0.18 | 1.52 |
| tDV_L_ | -0.12 | -0.38 to 0.14 | -0.29 | 0.35 | 5.60 |
| tDV_D_ | 0.04 | -0.19 to 0.27 | 0.10 | 0.72 | 4.53 |
| tSD_D_ | -0.11 | -0.40 to 0.17 | -0.27 | 0.42 | 6.57 |
|  |  |  |  |  |  |
| tDV: transformed dioptric volume  tSD_D_: transformed standard deviation of scene defocus  R: Right; U: Up; L: Left; D: Down  * indicated significance of p < 0.05 | | | | | |

Supplementary table 3. Multiple regression with all variables with doubled myopic defocus potency on refractive change over 1 year by ring analysis.

| Adjusted R^2^ = 0.35, F_16,50_ = 2.67, p = 0.01 | | | | | |
| --- | --- | --- | --- | --- | --- |
|  | Raw B value | 95% CI | Standardised  B value | p value | VIF |
| **Age** | **0.27*** | **0.03 to 0.51** | **0.63** | **0.03** | **1.50** |
| **Baseline M** | **0.08*** | **0.01 to 0.15** | **0.37** | **0.03** | **1.82** |
| Working distance | 0.02 | -0.02 to 0.05 | 0.23 | 0.30 | 3.56 |
| Time in front of desk | -0.09 | -0.34 to 0.16 | -0.10 | 0.45 | 1.40 |
| Parental myopia (ref no myopic parent) | | | | | |
| One myopic parent | -0.21 | -0.55 to 0.13 | -0.19 | 0.23 | 1.72 |
| Two myopic parents | 0.12 | -0.14 to 0.38 | 0.13 | 0.36 | 1.54 |
| Home size (ref Small) |  |  |  |  |  |
| Medium | 0.16 | -0.14 to 0.46 | 0.17 | 0.29 | 1.89 |
| Large | -0.10 | -0.41 to 0.21 | -0.11 | 0.51 | 1.98 |
| Time spent outdoors | 0.21 | -0.03 to 0.46 | 0.24 | 0.09 | 1.40 |
| tDV_2M5_ | 0.06 | -0.04 to 0.17 | 0.17 | 0.23 | 5.96 |
| tDV_2M10_ | 0.06 | -0.15 to 0.27 | 0.14 | 0.56 | 4.57 |
| tDV_2M15_ | -0.02 | -0.26 to 0.22 | -0.04 | 0.88 | 5.94 |
| tDV_2M20_ | -0.20 | -0.40 to 0.01 | -0.47 | 0.06 | 4.36 |
| tDV_2M25_ | -0.01 | -0.23 to 0.22 | -0.01 | 0.96 | 5.34 |
| tDV_2M30_ | 0.10 | -0.07 to 0.28 | 0.24 | 0.25 | 3.22 |
| tSD_D_ | -0.20 | -0.47 to 0.07 | -0.47 | 0.14 | 7.23 |
|  |  |  |  |  |  |
| tDV: transformed dioptric volume  tSD_D_: transformed standard deviation of scene defocus  2M indicates 2x myopic defocus potency  * indicated significance of p < 0.05 | | | | | |

Supplementary table 4. Multiple regression with all variables with doubled myopic defocus potency on refractive change over 1 year by quadrant analysis.

| Adjusted R^2^ = 0.19, F_14,50_ = 1.79, p = 0.08 | | | | | |
| --- | --- | --- | --- | --- | --- |
|  | Raw B value | 95% CI | Standardised  B value | p value | VIF |
| Age | 0.05 | -0.09 to 0.18 | 0.12 | 0.49 | 1.84 |
| Baseline M | 0.05 | -0.02 to 0.12 | 0.23 | 0.15 | 1.53 |
| Working distance | 0.00 | -0.03 to 0.04 | 0.05 | 0.81 | 2.63 |
| Time in front of desk | -0.13 | -0.40 to 0.14 | -0.14 | 0.34 | 1.28 |
| Parental myopia (ref no myopic parent) | | | | | |
| One myopic parent | -0.31 | -0.67 to 0.05 | -0.28 | 0.08 | 1.50 |
| Two myopic parents | 0.09 | -0.19 to 0.38 | 0.11 | 0.51 | 1.50 |
| Home size (ref Small) |  |  |  |  |  |
| Medium | 0.26 | -0.05 to 0.57 | 0.28 | 0.10 | 1.59 |
| Large | 0.01 | -0.29 to 0.30 | 0.01 | 0.97 | 1.47 |
| Time spent outdoors | 0.26 | -0.01 to 0.53 | 0.29 | 0.06 | 1.35 |
| tDV_2MR_ | 0.12 | -0.14 to 0.39 | 0.29 | 0.36 | 5.79 |
| tDV_2MU_ | 0.07 | -0.07 to 0.21 | 0.16 | 0.34 | 1.63 |
| tDV_2ML_ | -0.06 | -0.30 to 0.18 | -0.15 | 0.61 | 4.85 |
| tDV_2MD_ | 0.01 | -0.02 to 0.22 | 0.02 | 0.92 | 3.64 |
| tSD_D_ | -0.17 | -0.43 to 0.10 | -0.39 | 0.21 | 5.55 |
|  |  |  |  |  |  |
| tDV: transformed dioptric volume  tSD_D_: transformed standard deviation of scene defocus  R: Right; U: Up; L: Left; D: Down  2M indicates 2x myopic defocus potency  * indicated significance of p < 0.05 | | | | | |

Supplementary table 5. Descriptive statistics of the regional dioptric volume

|  | Median (D°°) | IQR | Range |
| --- | --- | --- | --- |
| DV_5_ | 0.56 | 0.35 to 0.90 | 0.22 to 4.07 |
| DV_10_ | 0.09 | 0.05 to 0.21 | -0.26 to 8.12 |
| DV_15_ | 0.14 | 0.04 to 0.44 | -0.41 to 3.84 |
| DV_20_ | 0.11 | -0.03 to 0.54 | -0.39 to 2.18 |
| DV_25_ | 0.05 | -0.16 to 0.57 | -0.96 to 2.27 |
| DV_30_ | -0.05 | -0.22 to 0.28 | -1.27 to 1.89 |
|  |  |  |  |
| DV_2M5_ | 0.54 | 0.34 to 0.88 | 0.22 to 3.50 |
| DV_2M10_ | 0.06 | 0.02 to 0.16 | -0.42 to 2.83 |
| DV_2M15_ | 0.11 | 0.01 to 0.34 | -0.64 to 3.82 |
| DV_2M20_ | 0.08 | -0.05 to 0.49 | -0.57 to 2.16 |
| DV_2M25_ | 0.01 | -0.26 to 0.58 | -1.05 to 2.21 |
| DV_2M30_ | -0.06 | -0.34 to 0.37 | -1.97 to 1.85 |
|  |  |  |  |
| DV_R_ | 0.16 | 0.10 to 0.30 | -0.01 to 1.23 |
| DV_U_ | -0.12 | -0.21 to -0.05 | -0.40 to 0.27 |
| DV_L_ | 0.33 | 0.11 to 0.75 | -0.37 to 2.05 |
| DV_D_ | 0.78 | 0.29 to 2.34 | -0.12 to 5.63 |
|  |  |  |  |
| DV_2MR_ | 0.14 | 0.08 to 0.28 | -0.07 to 1.23 |
| DV_2MU_ | -0.24 | -0.41 to -0.11 | -0.80 to 0.27 |
| DV_2ML_ | 0.30 | 0.07 to 0.71 | -0.78 to 2.02 |
| DV_2MD_ | 0.77 | 0.22 to 2.34 | -0.30 to 5.63 |
|  |  |  |  |
| DV: Dioptric volume  R: Right; U: Up; L: Left; D: Down  2M indicates 2x myopic defocus potency | | | |
